# Multi-omics Mendelian randomization integrating GWAS, eQTL, mQTL and pQTL data prioritizes mitochondrial gene *FXN* as a hypothesis−generating candidate for nephrolithiasis

**DOI:** 10.3389/fimmu.2026.1876576

**Published:** 2026-07-20

**Authors:** Yanting Wu, Shikai Ye, Ping Li, Zichi Wu, Zhiyuan Guo, Jun Bian, Ming Sheng, Dehui Lai

**Affiliations:** 1 Department of Urology, The Fifth Affiliated Hospital of Guangzhou Medical University, Guangzhou Medical University, Guangzhou, China; 2 Greater Bay Area Institute of Precision Medicine (Guangzhou), School of Life Sciences, Fudan University, Guangzhou, China; 3 School of Biological Sciences and Engineering, South China University of Technology, Guangzhou, China; 4 Department of Urology, The Sixth Affiliated Hospital of Jinan University, Dongguan, China; 5 Department of Urology, The Ninth People’s Hospital of Nanhai District, Foshan, China

**Keywords:** FXN, Mendelian randomization, mitochondrial dysfunction, multi-omics, nephrolithiasis, urolithiasis

## Abstract

**Background:**

Mitochondrial dysfunction is linked to urolithiasis, but causal genetic drivers remain unclear. We integrated multi-omics data using Mendelian randomization to identify mitochondrial-related genes causally associated with urolithiasis.

**Methods:**

We obtained mitochondrial methylation (mQTL), gene expression (eQTL), and protein abundance (pQTL) from respective quantitative trait locus (QTL) studies along with GWAS summary data for nephrolithiasis, ureterolithiasis and bladder calculus from the Million Veteran Program (discovery), with replication in FinnGen and UK Biobank. Summary-data-based Mendelian randomization (SMR) and colocalization were applied to infer causality.

**Results:**

Integrated analysis identified *FXN* as the leading candidate for nephrolithiasis. Genetically elevated circulating FXN protein was inversely associated with nephrolithiasis risk (OR 0.69, 95% CI 0.56–0.84). This protective effect was supported at the epigenetic level: *FXN* methylation at cg14656297 and cg13974534 correlated with lower nephrolithiasis risk. For ureterolithiasis, higher GRHPR protein levels were protective (OR 0.81, 95% CI 0.71–0.91). In a mouse kidney stone model, *Fxn* expression and frataxin protein levels were decreased, providing correlative *in vivo* support consistent with human genetic findings.

**Conclusion:**

This multi-omics MR study links mitochondrial genes, particularly FXN, to urolithiasis risk. Although colocalization evidence was weak (PP.H4 = 0.0239) and replication in independent cohorts was not statistically significant, the multi−omics consistency across methylation, expression, and protein levels prioritizes FXN as a hypothesis−generating candidate for further investigation. Because all QTL data are blood− or plasma−derived, this study provides blood/plasma QTL−based genetic prioritization rather than kidney−specific causal inference.

## 1 Introduction

Urolithiasis, commonly known as urinary stones, is a pathological condition characterized by the formation and aggregation of crystals within the urinary tract, including the kidneys, ureters, bladder, or urethra. This process primarily results from the supersaturation and precipitation of urinary solutes (1). As a highly prevalent global disease, it is characterized by a remarkably high recurrence rate of approximately 50%, posing a substantial lifelong burden on patients and healthcare systems (2). Clinical complications include renal colic from obstructing or migrating stones, which, if prolonged, can cause hydronephrosis, renal impairment, and eventually kidney failure (3). The context of these renal insults is particularly relevant given that renal tubular cells are among the most mitochondria-rich in the body, second only to cardiomyocytes (4).

Mitochondrial function in the nephron is essential for maintaining renal physiological homeostasis, and its dysfunction has been increasingly linked to the pathogenesis of urolithiasis (5–8). While oxalate is known to influence mitochondrial energy metabolism and calcium handling, its impact on membrane permeability or integrity appears to be more critical in the initiation of calcium oxalate microliths in the kidney (9). Although the mitochondrion is recognized as a key contributor to stone formation, the specific mitochondrial genes involved and their mechanistic roles remain poorly characterized. Mitochondrial dysfunction is known to trigger oxidative stress and NLRP3 inflammasome activation in kidney stone models, providing a biologically plausible context for *FXN*’s potential role; however, direct evidence linking *FXN* to these pathways is not provided by our data.

Mendelian randomization (MR), a powerful method that uses genetic instruments for robust causal inference between exposures and outcomes, typically requires individual-level data. Unlike observational studies, which are prone to confounding and reverse causality, MR provides more robust causal inference by leveraging genetic instruments (10, 11). As an extension that builds upon MR, summary-data-based Mendelian randomization (SMR) interrogates causal relationships by integrating summary statistics from genome-wide association studies (GWAS) and expression quantitative trait loci (eQTL) analyses (12, 13). SMR offers advantages over standard MR by efficiently addressing selection bias through improved statistical methods like support intervals and enabling not only the efficient use of public summary data but also the direct identification of potential causal genes. Urolithiasis has become a substantial public health burden. Nevertheless, the development of effective therapeutic strategies that prevent both stone formation and recurrence remains an unmet clinical challenge. Driven by the growing availability of large-scale GWAS and molecular QTL data, we can now investigate the causal role of mitochondrial-related genes in urolithiasis through their multifaceted molecular phenotypes: DNA methylation, gene expression, and protein abundance. Therefore, we conducted an integrated SMR and colocalization analysis to systematically evaluate the associations of mitochondrial gene methylation, expression, and protein abundance with urolithiasis risk, thereby offering mechanistic insights and highlighting potential therapeutic targets.

## 2 Study design and methods

### 2.1 Study design

**Figure 1:**
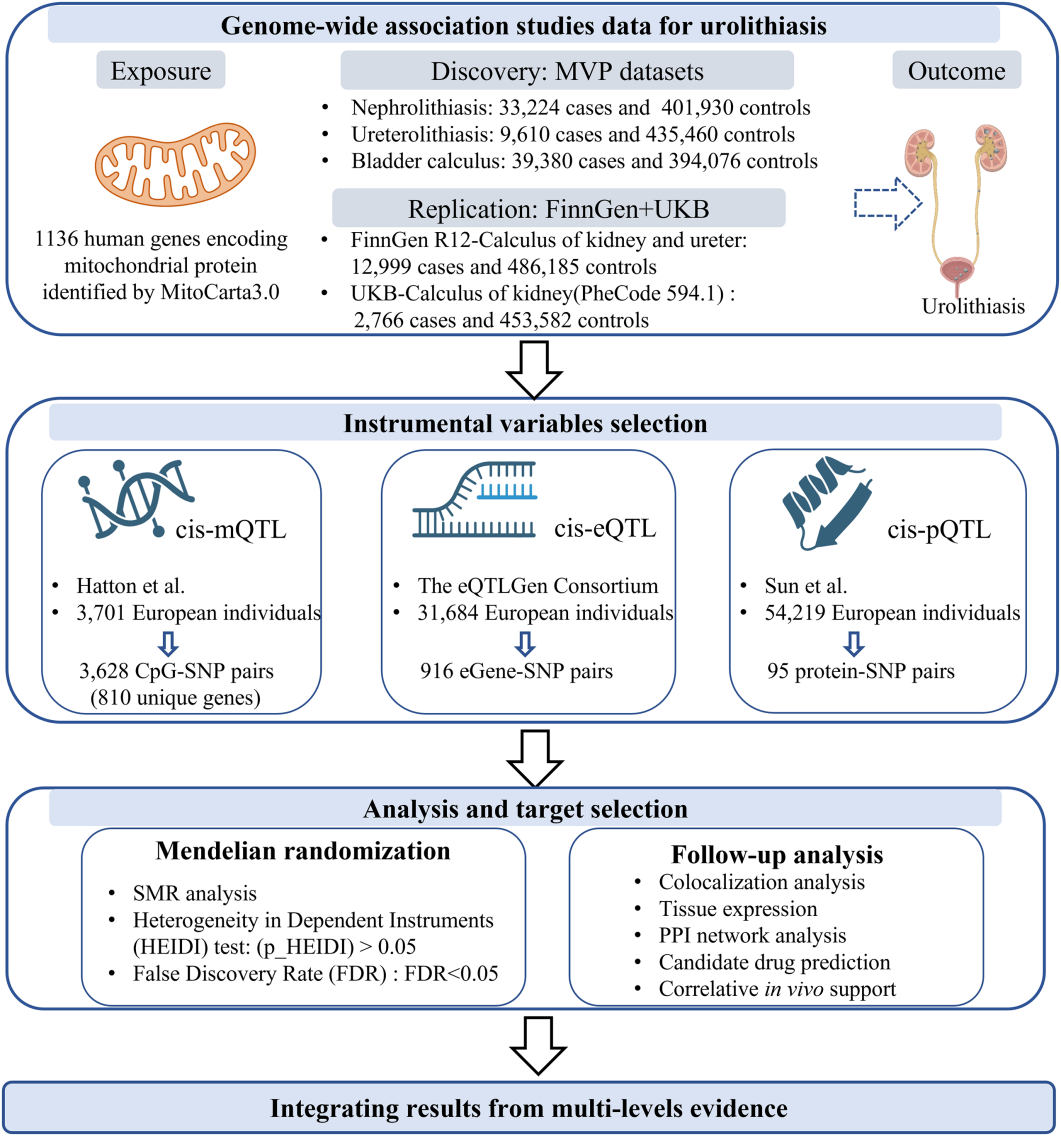
Study design. HEIDI, Heterogeneity in Dependent Instruments test; MVP, Million Veteran Program; QTL, quantitative trait loci; SMR, summary-based Mendelian randomization; SNP, single nucleotide polymorphisms; UKB, UK Biobank.

As outlined in **Figure 1**, the study began with the compilation of 1, 136 human mitochondrial-associated genes from the MitoCarta3.0 database, a resource providing comprehensive mitochondrial annotations and functional information (14). From this list, we selected mitochondrial-related instrumental variables based on quantitative trait loci (QTLs) for DNA methylation, gene expression, and protein abundance. The GWAS from Anurag Verma et al. (15). served as the primary discovery cohort. We subsequently performed summary-data-based Mendelian randomization (SMR) and heterogeneity in dependent instruments (HEIDI) tests to further evaluate potential causal relationships between exposures (mitochondrial gene) and outcomes (urolithiasis). Replication analyses were performed using the UK Biobank dataset (16) and the FinnGen dataset (17). To strengthen the causal evidence, we then employed colocalization analysis to determine whether the identified protein associations shared genetic causality with urolithiasis. Finally, by integrating SMR and follow-up analysis results, we aimed to identify mitochondrial-related genes implicated in urolithiasis pathogenesis.

### 2.2 Data sources

The set of mitochondrial-related genes analyzed in this study was curated from the authoritative MitoCarta3.0 inventory (http://www.broadinstitute.org/mitocarta). The GWAS data for urolithiasis including nephrolithiasis, ureterolithiasis and bladder calculus study outcomes were sourced from the NHGRI-EBI GWAS catalog (https://www.ebi.ac.uk/gwas/home) (18). Mitochondria-related QTL data were sourced from the following: mQTL data from a study by Hatton et al. (n=3, 701) (19), eQTL data from the eQTLGen consortium (n=31, 684) (20), and pQTL summary statistics from Sun et al. (n=54, 219) (21). Two independent datasets served as validation sets for urolithiasis genetic correlations. One was sourced from the FinnGen consortium (https://www.finngen.fi/en, Finngen-R12-Calculus of kidney and ureter), with 12, 999 cases and 486, 185 controls. The other was derived from the UK Biobank (https://www.ukbiobank.ac.uk/), specifically comprising 2, 766 nephrolithiasis cases and 453, 582 controls of European ancestry. Details of all GWAS datasets in this study are shown in **Data Sheet** - **Supplementary Table 1**.

### 2.3 SMR analysis and HEIDI test

SMR utilizes summary data from GWAS and cis-QTLs studies to evaluate the associations of mitochondrial gene methylation, expression, and protein abundance with the risk of urolithiasis. It also employs genetic variants (single-nucleotide polymorphisms, SNPs) as instrumental variables to assess causal relationships between exposures and outcomes (13). The SMR method increases statistical power compared to conventional MR by prioritizing cis-QTLs within a 1, 000 kb window of target genes and selecting the most significant association based on a locus-wide threshold (*P*-value < 5.0 × 10^-8^). The HEIDI test was applied to distinguish causal associations from those due to linkage disequilibrium (LD), using LD reference data from the 1000 Genomes Project. We performed SMR and HEIDI analyses using SMR v1.3.1 developed by the Yang Lab (https://yanglab.westlake.edu.cn) (13) and applied Benjamini-Hochberg correction (22) to control the false discovery rate (FDR) at 5%. Associations that met the criteria of FDR-adjusted *P*-values ≤ 0.05 and *P_HEIDI_* > 0.05 were advanced to colocalization analysis.

### 2.4 Colocalization analysis

We performed a Bayesian colocalization analysis (23) between mitochondrial-related cis-QTLs and urolithiasis using “coloc” R package (v5.2.3), aiming to identify robust causal relationships. We performed colocalization analysis only on probes with FDR < 0.05 that also passed the HEIDI test. Our analysis specifically focused on the posterior probability for hypothesis 4 (PP.H4), which posits that a single causal variant underlies the associations of both traits with all SNPs in the region. A PP.H4 ≥ 0.7 was considered as supportive evidence for colocalization, following previous recommendations.

### 2.5 Integrating results from multi-omics level of evidence

To comprehensively understand the associations between mitochondrial-related gene regulation and urolithiasis, we systematically integrated evidence across three tiers of gene regulation. Recognizing proteins as the functional end products of gene expression, we focused on identifying candidate genes whose associations with urolithiasis were significant at the protein abundance level. Consequently, the final candidate genes were categorized under a two-tiered classification system. Tier 1: Genes exhibited a significant association with urolithiasis at the protein level (*P_FDR_* ≤ 0.05, *P_HEIDI_*> 0.05); and were also significantly associated at both the DNA methylation and gene expression levels (*P_FDR_* ≤ 0.05, *P_HEIDI_*> 0.05). Tier 2: Genes significantly associated with urolithiasis at the protein abundance level (*P_FDR_* ≤ 0.05, *P_HEIDI_*> 0.05), as well as at either the DNA methylation or gene expression level (*P_FDR_* ≤ 0.05, *P_HEIDI_*> 0.05). We subsequently performed Bayesian colocalization analysis to elucidate the mechanisms underlying these multi-layer associations. The goal of these analyses was to determine the causal relationships between mitochondrial DNA methylation and gene expression, and between gene expression and protein abundance.

### 2.6 Expression in different tissues

We assessed the expression profiles of the identified causal genes across human tissues using data from The Human Protein Atlas (https://www.proteinatlas.org) (24) and the Genotype-Tissue Expression (GTEx) Portal (https://gtexportal.org/home/) (25).

### 2.7 PPI network analysis

Using the STRING database (https://cn.string-db.org/) (26), we constructed a protein-protein interaction (PPI) network from 36 previously identified causal genes. The network relevant to urolithiasis was restricted to “*Homo sapiens*” interactions with a confidence score threshold of 0.40, and disconnected proteins were hidden.

### 2.8 Candidate drug prediction

The Drug-Gene Interaction Database (DGIdb, www.dgidb.org) (27) classifies genes into druggability categories by integrating evidence from multiple source repositories, including DrugBank, PharmGKB, ChEMBL, Drug Target Commons, and the Therapeutic Target Database. To evaluate the therapeutic potential of our previously identified causal genes, we queried them against this resource. This assessment determined whether they were either known targets of existing drugs (reflecting drug-gene interactions) or classified as ‘potentially druggable’ based on their roles in specific pathways, molecular functions, or gene families (representing the druggable genome).

### 2.9 Mouse model of kidney stone

Specific pathogen-free (SPF) male C57BL/6 mice aged 6–8 weeks were purchased from Vital River Laboratory Animal Technology Co., Ltd. (Guangdong, China). These mice were housed in a SPF and temperature-controlled environment with a 12-hour light/dark cycle, and had unrestricted access to food and water. After a 7 days acclimation period, the mice were randomly divided into two groups, each consisting of 3 mice. To establish CaOx stone model, mice were intraperitoneally injected with glyoxylate (Gly, 100 mg/kg) for 10 days, while the control group received intraperitoneal injections of PBS. All experiments were carried out in accordance with the National Institutes of Health’s Guide for the Care and Use of Laboratory Animals. All experimental procedures received approval by the Institutional Animal Care and Use Committee of the Greater Bay Area Institute of Precision Medicine (Guangzhou) (Number: IPMGBA-23-0025).

### 2.10 RNA sequencing

Kidney tissues were collected from control mice and Gly−treated mice. Total RNA was extracted from the tissues using the Trizol method, followed by reverse transcription of 1 ;μg RNA into cDNA. The cDNA was subsequently ligated, amplified, purified, and quantified before library construction. High−throughput sequencing libraries were prepared and sequenced on a SURFseq5000 platform (Guangzhou Angte Biotechnology Co., Ltd.).

### 2.11 Histological staining

Histological analysis in the current study followed the methodology described in our prior work (28). In brief, fresh kidney tissue was fixed in 4% paraformaldehyde for 8 ;h at room temperature, dehydrated through a graded ethanol series, and paraffin−embedded. Sections of 3–5 ;µm thickness were prepared and stained with hematoxylin and eosin (HE), Masson’s trichrome, and Von Kossa methods. Histopathological evaluation of kidney injury, collagen deposition, and calcium oxalate crystal deposition was performed using a digital pathology scanner (Rui Bei Medical Technology). Quantification of fibrotic and crystal area was conducted using ImageJ software.

### 2.12 Immunoblotting

Kidney tissue proteins were extracted using RIPA buffer supplemented with protease and phosphatase inhibitors. Total protein was separated by SDS-PAGE and transferred onto 0.22 ;μm polyvinylidene difluoride (PVDF) membranes. The membranes were incubated overnight at 4°C with primary antibodies against Frataxin and β−Actin, followed by incubation with appropriate secondary antibodies for 1 ;h at room temperature. Protein signals were detected using enhanced chemiluminescence (ECL) reagent and visualized with a ChemiDoc XRS imaging system (Bio−Rad). The protein abundance was quantified using the Image J software.

## 3 Results

### 3.1 Mitochondrial gene methylation and urolithiasis

**Figure 2:**
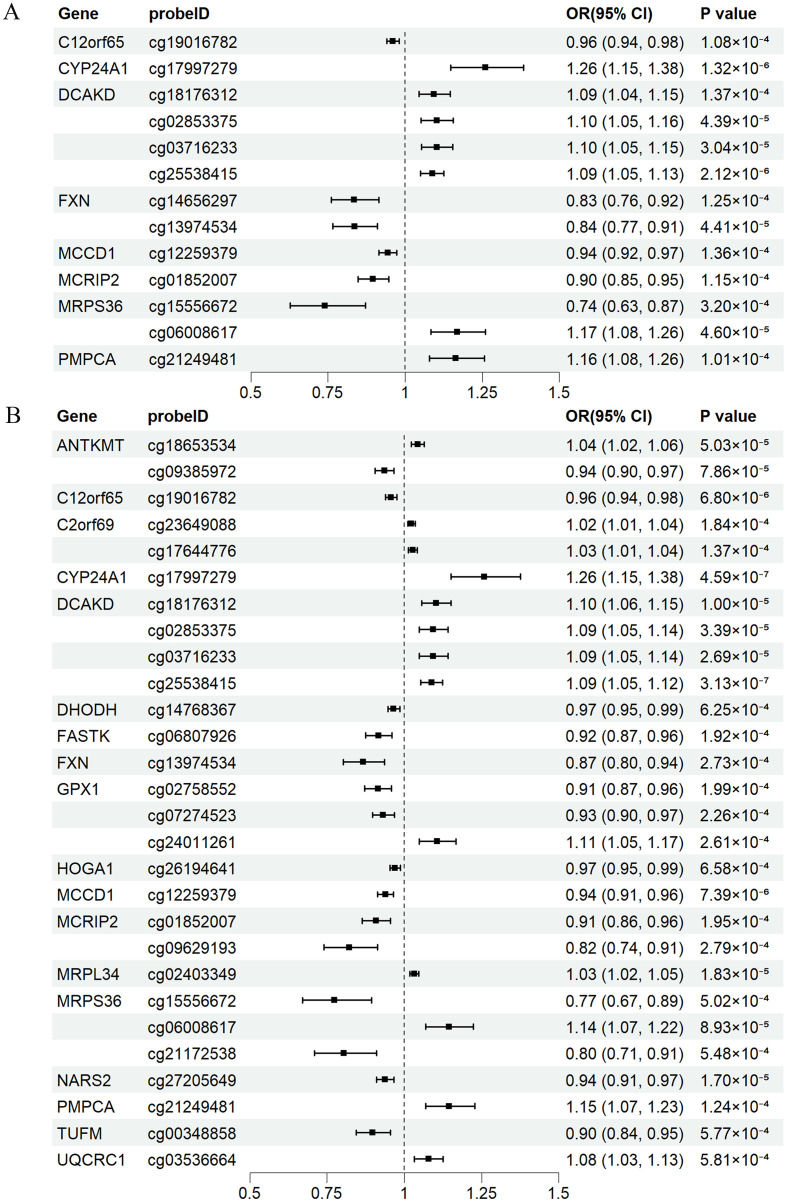
Associations of genetically predicted mitochondrial gene methylation with urolithiasis in Mendelian randomization analysis. **(A)** Nephrolithiasis. **(B)** Bladder calculus.

This study employed SMR analysis to examine the potential causal link between mitochondrial gene methylation and urolithiasis. The β coefficient served as the causal estimate, and the corresponding odds ratio (OR) for a one-SD change in methylation level was utilized to interpret its magnitude. After FDR correction (*p* ≤ 0.05) and the HEIDI test (*p*_HEIDI > 0.05), we identified 13 CpG sites mapped to 8 unique genes (*CYP24A1, DCAKD, FXN, MRPS36, PMPCA, C12orf65, MCRIP2*) associated with nephrolithiasis (**Figure 2A**), and 28 sites located mapped to 18 unique genes (*ANTKMT, C2orf69, C12orf65, CYP24A1, DCAKD, DHODH, FASTK, FXN, GPX1, HOGA1, MCCD1, MCRIP2, MRPL34, MRPS36, NARS2, PMPCA, TUFM, UQCRC1*) were associated with bladder calculus (**Figure 2B**). Among these, seven CpG sites across four unique genes showed strong evidence of colocalization (PP.H4 > 0.70), specifically within *MRPS36* (cg06008617, cg15556672, cg21172538), *MCRIP2* (cg01852007, cg09629193), *NARS2* (cg27205649), and *GPX1* (cg02758552) (**Data Sheet** - **Supplementary Table 2**). Notably, we observed heterogeneity in the direction of effect estimates among CpG sites within the same gene. For example, in *MRPS36*, CpG sites cg15556672 (OR 0.77, 95% CI 0.67–0.89) and cg21172538 (OR 0.80, 95% CI 0.71–0.91) showed a negative association with urolithiasis, whereas cg06008617 (OR 1.14, 95% CI 1.07–1.22) showed a positive association. Additionally, the associations between mitochondrial genes and urolithiasis were replicated in the FinnGen R12 database and UK Biobank database (**Data Sheet** - **Supplementary Tables 3**, **4**).

### 3.2 Mitochondrial gene expression and urolithiasis

**Figure 3:**
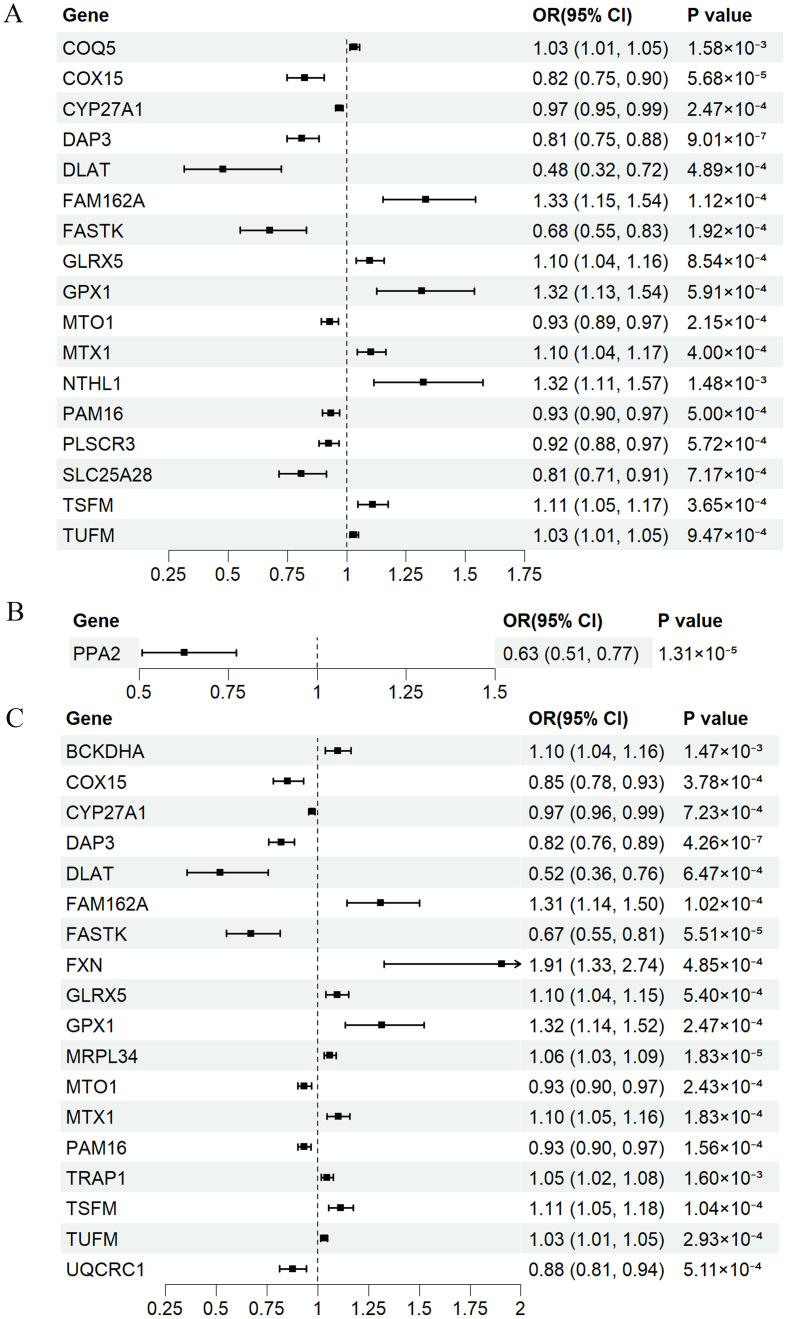
Associations of genetically predicted mitochondrial gene expression with urolithiasis in Mendelian randomization analysis. **(A)** Nephrolithiasis. **(B)** Ureterolithiasis. **(C) **Bladder calculus.

Following SMR analysis with FDR correction (p ≤ 0.05) and the HEIDI test (*P_HEIDI_* > 0.05), 23 unique genes were identified as significantly associated with urolithiasis (**Data Sheet** - **Supplementary Table 2**). The subtype-specific associations were as follows: 17 genes (*DAP3, COX15, FAM162A, FASTK, MTO1, CYP27A1, TSFM, MTX1, DLAT, PAM16, PLSCR3, GPX1, SLC25A28, GLRX5, TUFM, NTHL1, COQ5*) with nephrolithiasis (**Figure 3A**), one with ureterolithiasis (**Figure 3B**), and 18 genes (*DAP3, MRPL34, FASTK, FAM162A, TSFM, PAM16, MTX1, MTO1, GPX1, TUFM, COX15, FXN, UQCRC1, GLRX5, DLAT, CYP27A1, BCKDHA, TRAP1*) with bladder calculus (**Figure 3C**). Further colocalization analysis revealed that increased expression of the mitochondrial gene *COX15* (OR 0.82, 95% CI 0.75–0.90, PP.H4 = 0.93), *DAP3* (OR 0.81, 95% CI 0.75–0.98, PP.H4 = 0.97) and *DLAT* (OR 0.48, 95% CI 0.32–0.72, PP.H4 = 0.71) were closely associated with a lower risk of nephrolithiasis. Similarly, higher expression of *PPA2* (OR 0.63, 95% CI 0.51–0.77, PP.H4 = 0.71) was closely associated with a lower risk of ureterolithiasis. For bladder calculus, elevated expression of *DAP3* (OR 0.82, 95% CI 0.76–0.89, PP.H4 = 0.97) and *DLAT* (OR 0.52, 95% CI 0.36–0.76, PP.H4 = 0.74) was associated with a decreased risk, whereas higher *TSFM* expression (OR 1.11, 95% CI 1.05–1.18, PP.H4 = 0.72) was associated with an increased risk (**Data Sheet** - **Supplementary Table 5**). Regarding *FXN* and bladder calculus, the association signals were directionally inconsistent across molecular layers: methylation at cg13974534 was protective (OR 0.83, 95% CI 0.76–0.92), whereas higher *FXN* expression was associated with increased risk (OR 1.91, 95% CI 1.33–2.74). Given this complexity and the lack of colocalization or replication, these FXN-related bladder calculus findings should be considered exploratory and are not further prioritized. Additionally, the associations between mitochondrial genes and urolithiasis were replicated in the FinnGen R12 database and UK Biobank database (**Data Sheet** - **Supplementary Tables 6**, **7**).

### 3.3 Mitochondrial protein and urolithiasis

**Figure 4:**
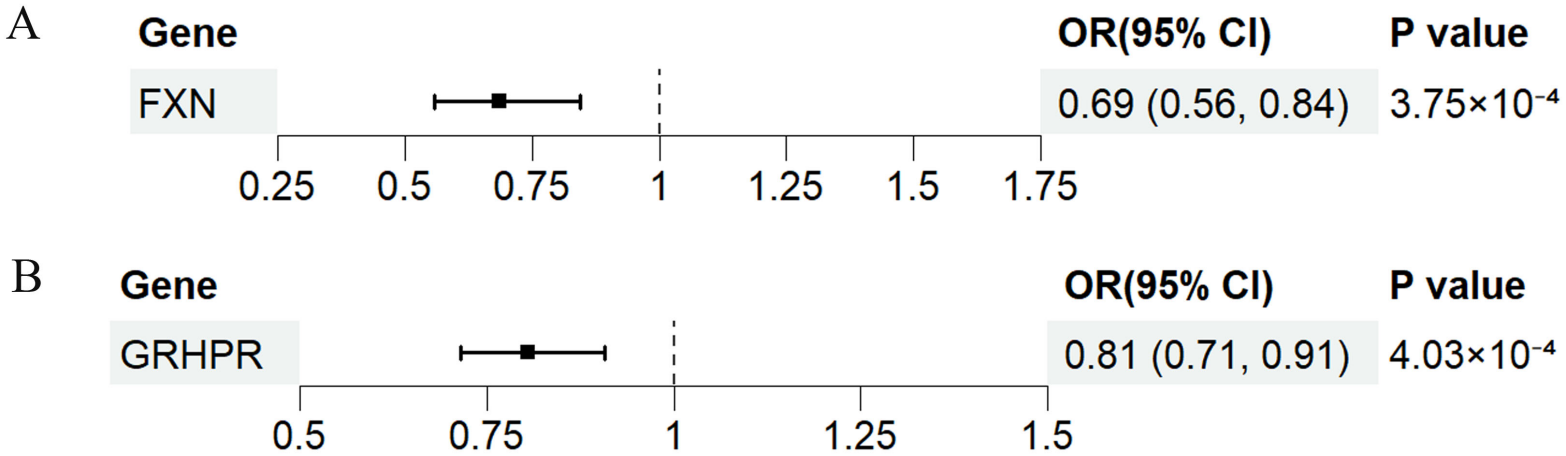
Associations of genetically predicted mitochondrial protein with urolithiasis in Mendelian randomization analysis. **(A)** Nephrolithiasis. **(B)** Ureterolithiasis.

A total of 11, 5, and 10 mitochondrial proteins were initially identified as being associated with the risk of nephrolithiasis, ureterolithiasis, and bladder calculus, respectively (*P_SMR_* < 0.05 and *P_HEIDI_* > 0.05). After FDR correction (p ≤ 0.05), one significant protein (FXN) for nephrolithiasis (**Figure 4A**) and one (GRHPR) for ureterolithiasis (**Figure 4B**) remained. Subsequent colocalization analysis further indicated that a higher level of GRHPR (OR 0.81, 95% CI 0.71–0.91, PP.H4 = 0.80) was associated with a reduced risk of ureterolithiasis (**Data Sheet** - **Supplementary Table 8**). In contrast, the colocalization support for the FXN protein association with nephrolithiasis was weak (PP.H4 = 0.0239, far below the prespecified threshold of 0.7), indicating that FXN protein abundance and nephrolithiasis do not share a single causal variant in the current data (**Data Sheet** - **Supplementary Table 8**). Furthermore, the *FXN* pQTL finding was not statistically replicated in FinnGen or UK Biobank, although the effect direction was consistent across both replication datasets (**Data Sheet** - **Supplementary Tables 9**, **10**).

### 3.4 Integrating evidence from multi-omics levels

We reasoned that proteins, as the functional end products of gene expression, would provide a robust foundation for identifying candidate genes. This approach identified *FXN*, whose protein expression is inversely associated with nephrolithiasis risk. This protective role is further supported at the epigenetic level, where two *FXN* CpG sites cg13974534 (OR 0.83, 95% CI 0.76–0.92) and cg14656297 (OR 0.84, 95% CI 0.77–0.91) were also negatively associated with the nephrolithiasis. Although the SMR analysis for mitochondrial gene expression yielded an FDR-corrected p-value (0.050443) marginally above the significance threshold (**Data Sheet** - **Supplementary Table 5**), the nominally significant SMR p-value (*P_SMR_* = 1.74×10^-3^) in conjunction with a non-significant HEIDI test (HEIDI > 0.05) supports a suggestive association between *FXN* gene expression and nephrolithiasis. Thus, by integrating multi−omics evidence, we identify *FXN* as the leading Tier ;2 candidate for nephrolithiasis, characterized by a consistent inverse relationship between its protein abundance and disease risk, supported by methylation and nominally significant expression evidence.

For bladder calculus, the multi−omics evidence for *FXN* was less consistent. A suggestive SMR association for protein abundance (*P_SMR_* = 8.75×10^-4^, *P_HEIDI_* > 0.05) with an FDR−corrected p−value of 0.081 was supported by significant methylation at cg13974534 (OR 0.83, 95% CI 0.76–0.92) and by *FXN* gene expression (OR 1.91, 95% CI 1.33–2.74; *P_FDR_*≤ 0.05). However, the direction of effect was inconsistent: methylation was protective (OR < 1) whereas higher expression was associated with increased risk (OR > 1) (**Data Sheet** - **Supplementary Table 2**). Given this directional complexity and the lack of colocalization or replication, the bladder calculus findings for *FXN* should be considered exploratory and are not further prioritized.

Importantly, the multi−omics evidence for *FXN* in nephrolithiasis is convergent but preliminary. No single causal variant links all three molecular layers (methylation, expression, and protein abundance) to disease risk in the current data. Therefore, *FXN* should be considered a prioritized hypothesis−generating candidate rather than a demonstrated causal protective gene.

### 3.5 FXN expression in human tissue

**Figure 5:**
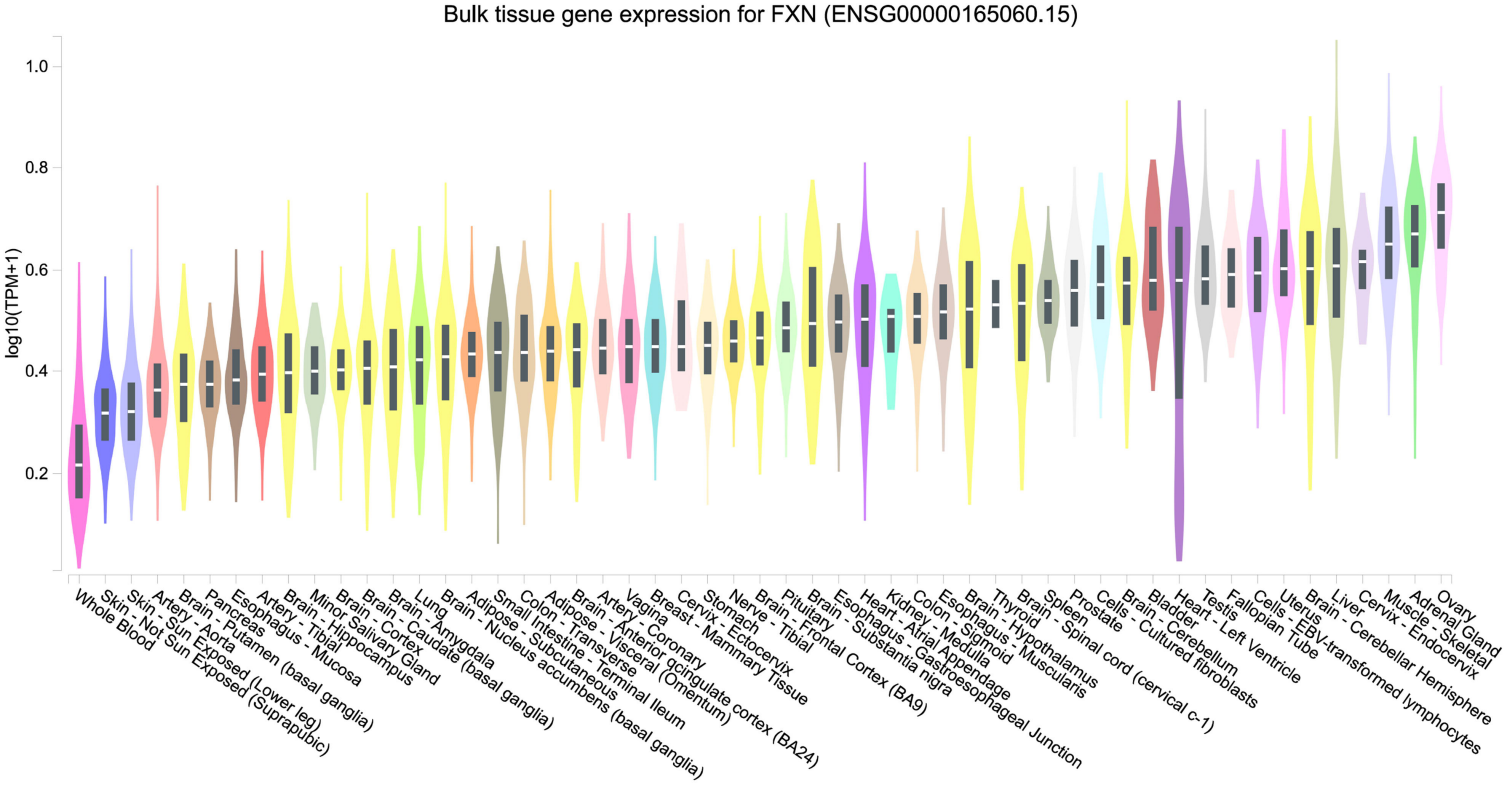
The expression of FXN in human tissues.

We evaluated the expression of putative causal genes, especially *FXN*, in tissues relevant to urolithiasis (kidney, ureter, and bladder) using data from the Human Protein Atlas and the GTEx portal. We found moderate to high expression levels in these organs (**Figure 5**, **Data Sheet** - **Supplementary Table 11**). Given that FXN protein abundance is inversely associated with disease risk, its robust expression at the primary disease sites positions FXN as a promising candidate for further investigation.

### 3.6 Protein-protein interaction and pathway enrichment analysis

**Figure 6:**
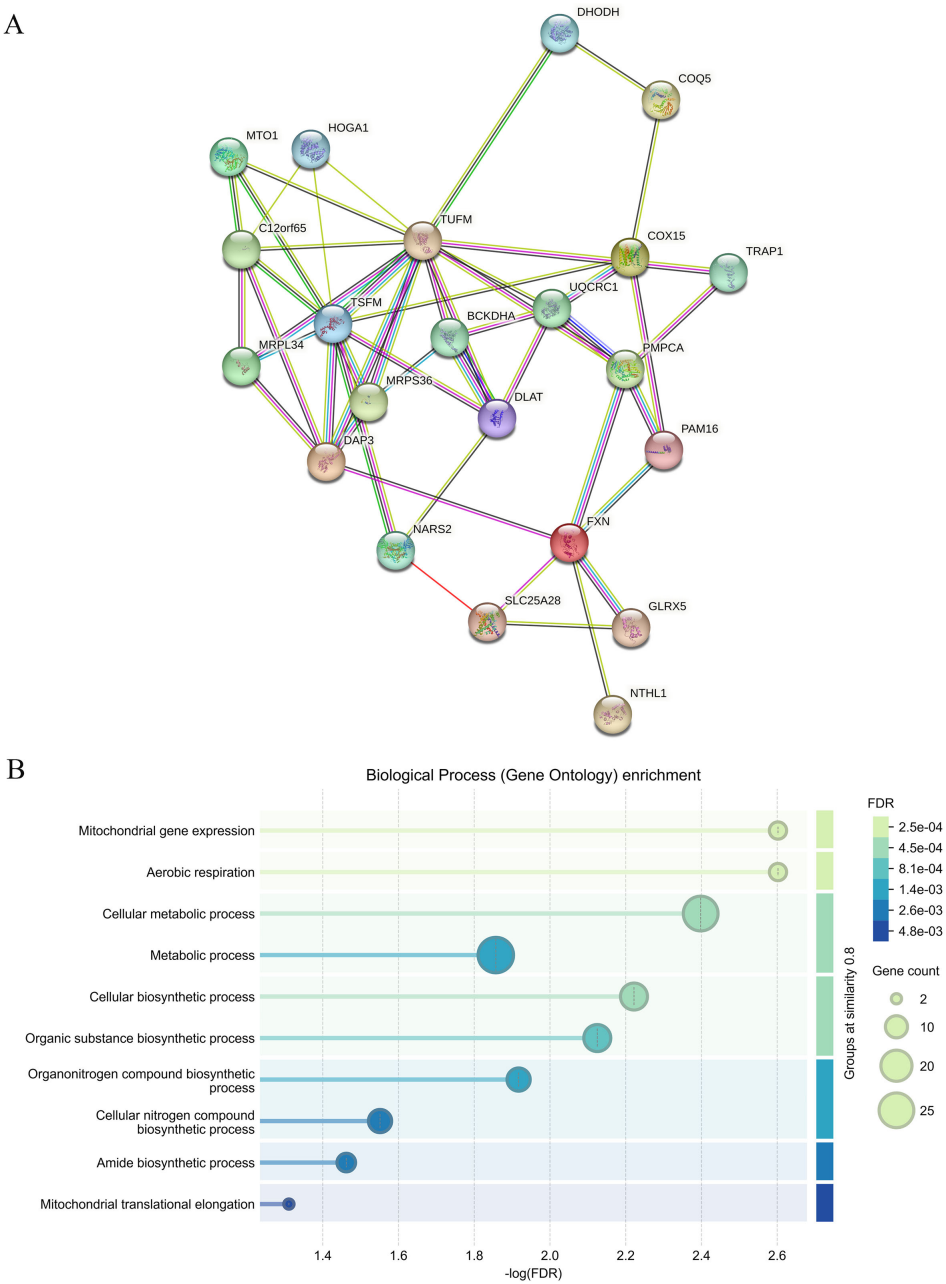
Candidate causal genes network analysis and pathway enrichment. **(A)** STRING PPI network of candidate causal genes. **(B)** Pathway enrichment of candidate causal genes based on the Gene Ontology (GO) biological process category.

To elucidate the interactions among mitochondria-related proteins and their structural and functional relevance in urolithiasis, we constructed protein-protein interaction (PPI) networks for the 36 mitochondrial proteins identified as significant in the SMR analysis. The resulting network, generated with an interaction confidence score threshold of 0.4 and exhibiting a highly significant PPI enrichment p-value (< 1.0 × 10^-16^), revealed key functional modules. Notably, TUFM, TSFM, and FXN formed a tightly interconnected cluster, suggesting their coordinated involvement in metabolic and disease-relevant pathways (**Figure 6A**, **Data Sheet** - **Supplementary Table 12**). Consistent with this, pathway enrichment analysis demonstrated that these significant genes were primarily associated with biological processes related to aerobic respiration and metabolic regulation (**Figure 6B**; complete Gene Ontology results are provided in **Data Sheet** - **Supplementary Table 13**).

### 3.7 Candidate drug prediction

**Figure 7:**
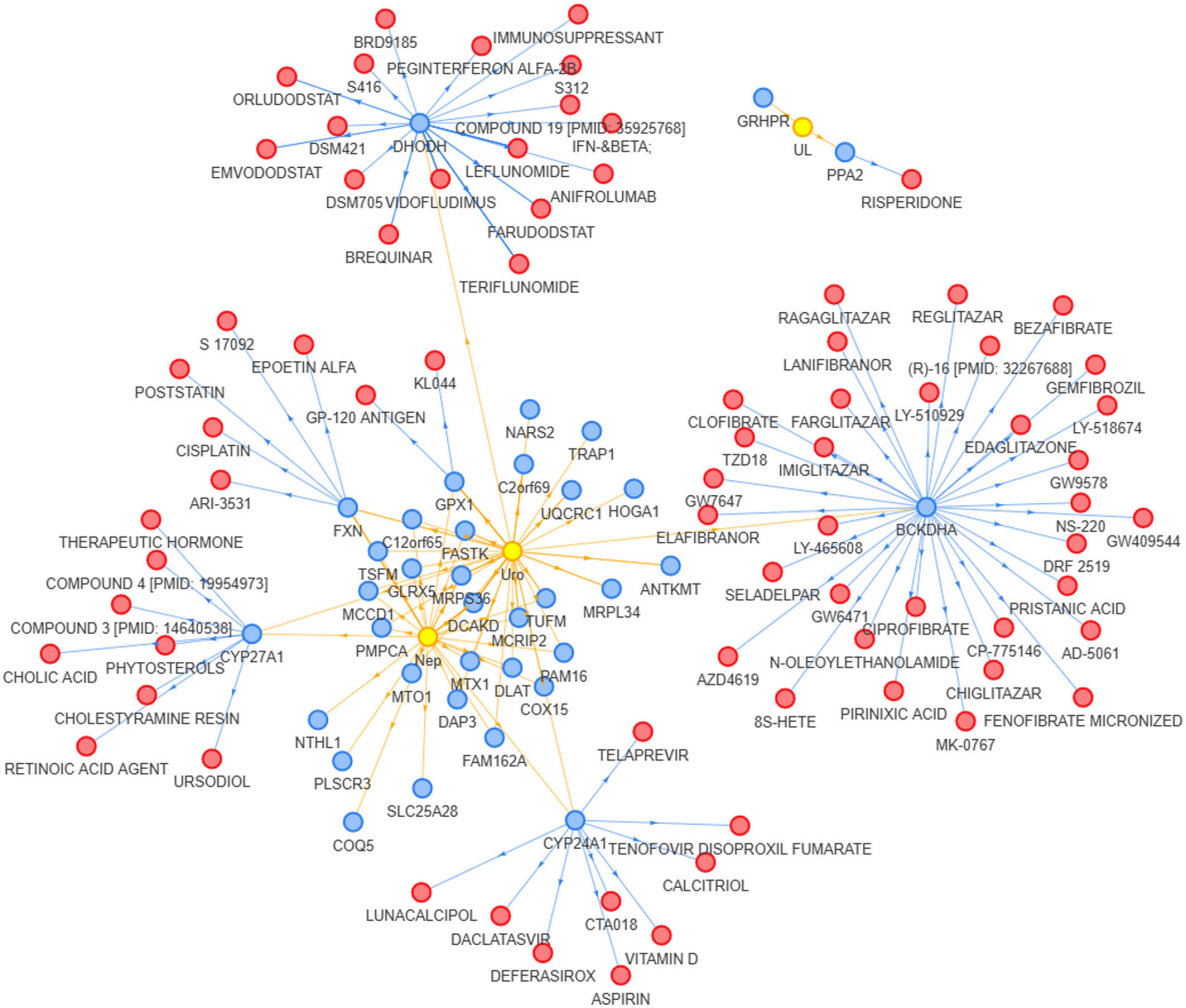
Drug interaction results for candidate causal genes based on DGIdb database. Yellow dot represents disease, blue dot represents causal gene, red dot represents drug.

We screened the previously identified causal genes using the DGIdb database to assess their therapeutic potential. This analysis yielded 75 drugs associated with urolithiasis, 21 of which are approved, that target these causal genes (**Data Sheet** - **Supplementary Table 14**). Among them, the genes with the most drug associations were *BCDKHA* (33 drugs), *DHODH* (17 drugs), *CYP24A1* (9 drugs), *CYP27A1* (8 drugs), and *FXN* (5 drugs) (**Figure 7**).

### 3.8 Decreased Fxn expression in a mouse kidney stone model provides preliminary correlative support

**Figure 8:**
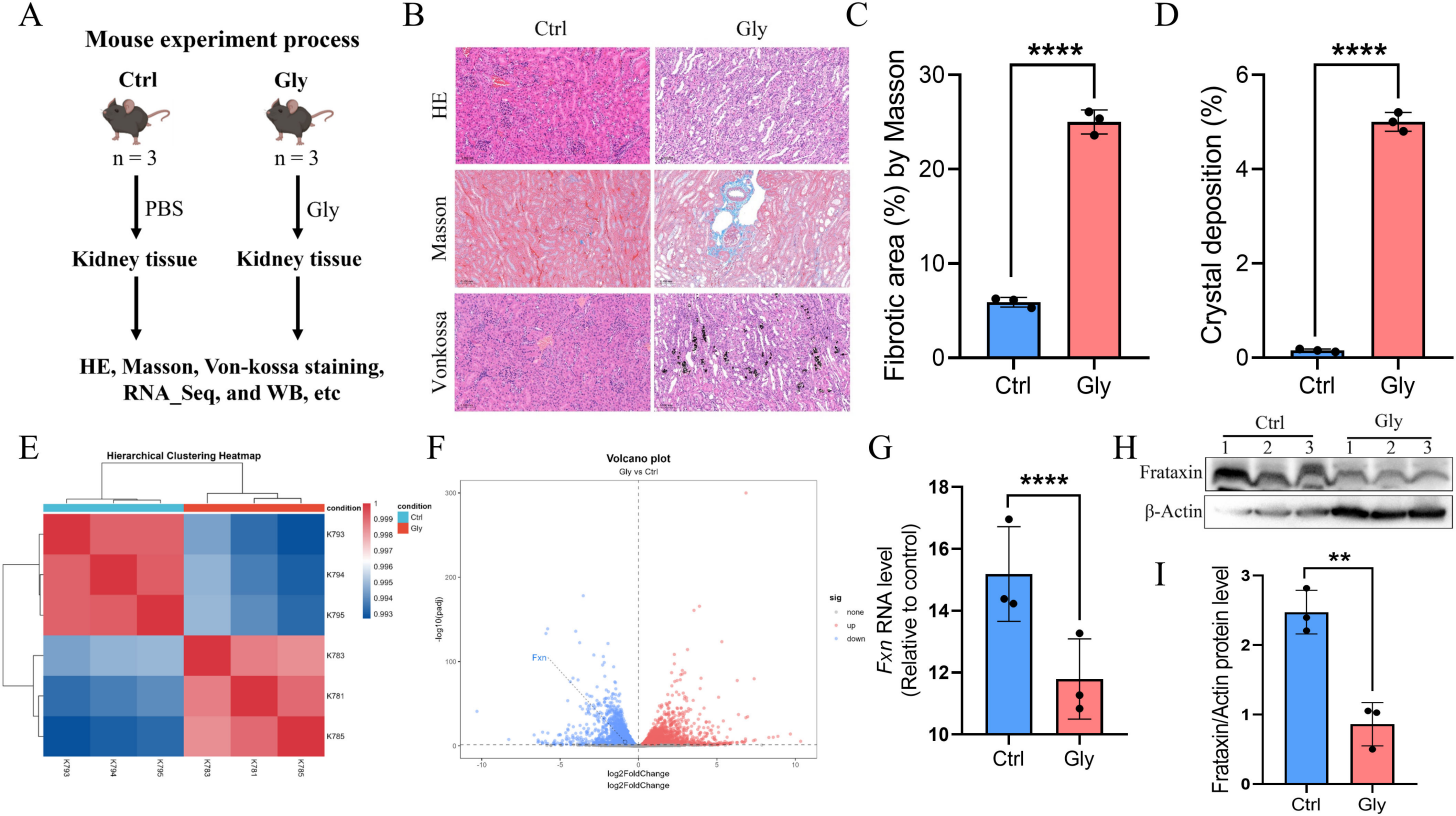
The level of Fxn is decreased in mice with kidney stones. **(A)** The experimental design. **(B)** Representative images of HE staining, Masson staining and Von-kossa staining of kidney tissues from control mice and Gly mice. Scale bar, 100 μm. **(C)** Quantification of fibrotic area was conducted using ImageJ software. **(D)** Quantification of crystal area was conducted using ImageJ software. **(E)** The sample correlation of RNA-seq. **(F)** Volcano map of DEGs in kidney tissues from control mice and Gly mice based on the RNA- seq. **(G)** The relative gene expression of *Fxn* based on the RNA- seq. **(H)** The protein abundance of frataxin. β-actin was used as a control. **(I)** Relative quantification of protein expression of frataxin. Data are presented as the mean ± SD of 3 samples. Statistical analyses were performed with one-way ANOVA, **p < 0.01, ****p < 0.0001.

**T**o explore the *in vivo* relevance of the human genetic findings, we induced kidney stones in mi**ce** using glyoxylate and confirmed stone formation by histopathological examinations, including HE staining, Masson staining, and Von Kossa staining (**Figures 8B–D**). RNA−seq analysis of renal tissues subsequently revealed significantly reduced Fxn expression in the glyoxylate−induced group compared to normal controls (**Figures 8E–G**), which was further validated by decreased frataxin protein levels using Western blot (**Figures 8H, I**). These data demonstrate that reduced Fxn expression is associated with nephrolithiasis in mice. However, because this experiment was purely observational (no genetic or pharmacological manipulation of *Fxn*), it does not establish a causal relationship. Reduced *Fxn* expression could be a downstream consequence of crystal−induced injury, oxidative stress, inflammation, or tubular damage rather than a primary protective mechanism. Therefore, this experiment provides preliminary correlative *in vivo* evidence supporting the protective direction inferred from human Mendelian randomization, but it should not be interpreted as validation of causality.

## 4 Discussion

Urolithiasis, a common urological disorder characterized by the formation of mineral crystals in the urinary tract, has recently been linked to mitochondrial gene mutations and functional impairments. As the cellular powerhouses, mitochondria play a critical role in energy metabolism, and their dysfunction can lead to oxidative stress and metabolic disturbances, such as abnormal calcium and oxalate handling in renal cells. These alterations may promote crystal formation and growth, contributing to stone development (5). Emerging evidence suggests that certain hereditary stone syndromes involve mtDNA mutations, highlighting the potential role of mitochondrial integrity in urolithiasis pathogenesis (7, 29–31). In this study, we aimed to elucidate the genetic underpinnings of mitochondrial dysfunction in relation to urolithiasis by employing an integrated multi-omics approach, which included SMR and colocalization analyses. We systematically gathered summary-level data on mitochondrial gene methylation, expression, and protein abundance from molecular quantitative trait loci studies, and assessed genetic associations with nephrolithiasis, ureterolithiasis, and bladder calculus using data from the Million Veteran Program, UK Biobank, and FinnGen study.

Our findings reveal significant associations between specific mitochondrial genes, particularly *FXN*, and the risk of nephrolithiasis and bladder calculus. Genetically elevated circulating FXN protein was inversely associated with nephrolithiasis risk (OR 0.69, 95% CI 0.56–0.84). This protective effect was supported at the epigenetic level: *FXN* methylation at cg14656297 and cg13974534 correlated with lower nephrolithiasis risk. Although the SMR analysis for *FXN* gene expression yielded an FDR-corrected p-value marginally above the significance threshold (p = 0.050443), the nominally significant SMR p-value (1.74×10^-3^) together with a non-significant HEIDI test supports a suggestive association. Frataxin, encoded by *FXN*, is a mitochondrial protein crucial for iron-sulfur cluster biogenesis and mitochondrial iron homeostasis (32–34). Its deficiency disrupts ATP synthesis and diminishes oxygen consumption efficiency (35–37). Given frataxin’s established role in reducing oxidative stress, it is plausible that *FXN* could modulate inflammatory pathways such as NLRP3 inflammasome activation, which has been implicated in kidney stone formation (5). However, the current study does not directly assess inflammasome activity, immune cell infiltration, or inflammatory cytokines. Therefore, this proposed mechanism remains speculative and should be tested in future studies using appropriate *in vitro* or *in vivo* models (e.g., *Fxn*−knockout renal tubular cells or stone−prone mice with immune readouts).

A Bayesian colocalization analysis did not support a shared causal variant for FXN protein abundance and nephrolithiasis (PP.H4 ;< ;0.7). While this negative result could be interpreted as challenging the causality of *FXN*, we believe it primarily reflects a tissue mismatch between the QTL data (derived from peripheral blood) and the disease pathology (kidney). Gene regulation, including DNA methylation, expression, and protein levels, is known to be highly tissue−specific. Cis−regulatory variants identified in blood may not capture the regulatory architecture of the kidney, where stone formation occurs. Therefore, the absence of colocalization does not negate the biological relevance of *FXN* but rather underscores the need for kidney−derived molecular QTL resources, such as GTEx kidney cortex and NephQTL. Until such data become available, the multi−omics consistency across methylation, expression (nominally), and protein levels remains the strongest evidence supporting *FXN* as a candidate gene. Alternatively, we cannot exclude the possibility that the observed association is driven by linkage disequilibrium with another causal variant, or that horizontal pleiotropy contributes to the SMR signal.

The mouse experiment was designed only as a phenotypic validation, not as an interventional causality test. The observed decrease in *Fxn* expression could be a consequence of crystal−induced injury rather than a primary protective mechanism. Future loss−of−function (e.g., tubule−specific *Fxn* knockout) or gain−of−function (e.g., frataxin overexpression or supplementation) studies in stone−prone models are required to establish whether *FXN* plays a genuine causal protective role. Therefore, while the directionality aligns with the human MR findings, these data should be considered hypothesis−generating. Definitive evidence of causality and therapeutic potential will come from future loss−of−function (e.g., tubule−specific Fxn knockout) or gain−of−function (e.g., frataxin overexpression or supplementation) studies in stone−prone models.

The strengths of our research methods lie in the robust integration of multi-omics data, which allows for a comprehensive exploration of the genetic underpinnings of mitochondrial dysfunction in relation to urolithiasis. By employing SMR and colocalization approaches, we inferred potential causal relationships between mitochondrial-related genes and various forms of kidney stones, thereby generating hypotheses for future testing. The utilization of summary-level data from large-scale studies, such as the Million Veteran Program, UK Biobank, and FinnGen, not only bolstered the statistical power of our analyses but also facilitated the replication of results across diverse populations.

Several additional limitations must be emphasized regarding the *FXN* finding. First, the colocalization posterior probability for FXN protein abundance with nephrolithiasis was only 0.0239, well below our threshold for supporting a shared causal variant. Second, the FXN pQTL association did not replicate significantly in FinnGen or UK Biobank, although directionally consistent. Therefore, the current data do not support a causal relationship at the genetic level. The multi-omics consistency across methylation, expression (nominally), and protein levels should be interpreted as hypothesis-generating, not as evidence of causality. More broadly, tissue specificity of QTL resources is a major limitation. The mQTL and eQTL data are derived from peripheral blood (19, 20), and the pQTL data reflect circulating plasma protein abundance (21). However, nephrolithiasis is a disease of the kidney and urinary tract, where mitochondrial function in renal tubular epithelial cells is central to pathogenesis. Although we show that *FXN* is expressed in human kidney and bladder tissues (**Figure 5**), this does not establish that the cis−regulatory variants identified in blood operate similarly in renal cells. Gene regulation is highly tissue−specific, and QTL effects often differ between blood and target organs. Therefore, our findings should be interpreted as blood/plasma QTL−based genetic prioritization rather than kidney−specific causal inference. Currently, large−scale, publicly available kidney−specific mQTL, eQTL, and pQTL resources with sufficient statistical power for SMR analysis are not available for European ancestry populations. Resources such as NephQTL and GTEx kidney cortex have limited sample sizes. Until such data become available, the current results are hypothesis−generating and should guide future tissue−specific investigations.

Differences in phenotype definitions across cohorts may partly explain the lack of statistical replication for *FXN*. The discovery MVP cohort allowed separate analyses for nephrolithiasis, ureterolithiasis, and bladder calculus, while FinnGen used a combined endpoint of “calculus of kidney and ureter” (R12) that includes both nephrolithiasis and ureterolithiasis. UK Biobank defined nephrolithiasis using self−report and hospital codes but did not distinguish upper vs. lower urinary tract stones with the same granularity as MVP. Thus, the absence of significant replication could reflect genuine weak evidence, phenotype heterogeneity, or both. Given the consistent direction of effect across cohorts but lack of statistical significance, we cannot rule out that the weak colocalization and limited statistical power in replication cohorts (especially UK Biobank with only 2, 766 cases) contribute to the non−replication. Therefore, the current evidence for *FXN* remains hypothesis−generating, and replication in larger, more homogeneously phenotyped cohorts is needed.

In summary, this multi-omics MR study prioritizes *FXN* as a hypothesis-generating candidate for nephrolithiasis based on consistent multi-omics signals (methylation, nominal expression, and protein levels), although colocalization evidence was inconclusive (PP.H4 = 0.0239), and replication in independent cohorts did not reach statistical significance. The weak colocalization may be due at least in part to tissue mismatch (blood-derived QTLs vs. kidney target organ). Because all QTL data are blood- or plasma-derived, the findings do not establish kidney-specific causality. Instead, this study provides blood-based genetic prioritization of *FXN* for nephrolithiasis. Given the lack of robust colocalization and replication, *FXN* should not be interpreted as a demonstrated causal protective gene but rather as a prioritized candidate that requires functional validation (e.g., kidney-specific Fxn knockout or overexpression studies) to establish causality and therapeutic potential.

## Data Availability

The data presented in the study are deposited in the zenodo repository, accession number 10.5281/zenodo.20200820.
